# Bortezomib, lenalidomide, and dexamethasone (VRd) followed by autologous stem cell transplant for multiple myeloma

**DOI:** 10.1038/s41408-018-0147-7

**Published:** 2018-11-08

**Authors:** M. Hasib Sidiqi, Mohammed A. Aljama, Irbaz Bin Riaz, Angela Dispenzieri, Eli Muchtar, Francis K. Buadi, Rahma Warsame, Martha Q. Lacy, David Dingli, Nelson Leung, Wilson I. Gonsalves, Prashant Kapoor, Taxiarchis V. Kourelis, William J. Hogan, S. Vincent Rajkumar, Shaji K. Kumar, Morie A. Gertz

**Affiliations:** 1 Division of Hematology, Department of Internal Medicine, Mayo Clinic Rochester, Rochester, MN USA; 2 Division of Nephrology, Department of Internal Medicine, Mayo Clinic Rochester, Rochester, MN USA

## Abstract

We retrospectively reviewed all patients (*n* = 243) receiving bortezomib, lenalidomide, and dexamethasone (VRd) induction followed by autologous stem cell transplantation (ASCT) for multiple myeloma at the Mayo Clinic between January 2010 and April of 2017. Median age was 61 (interquartile range, 55–67) with 62% of patients being male. High-risk cytogenetic abnormalities (HRA) were present in 34% of patients. A total of 166 (68%) patients received some form of maintenance/other therapy post transplant (no maintenance (NM, *n* = 77), lenalidomide maintenance (LM, *n* = 108), bortezomib maintenance (BM, *n* = 39), and other therapy (OT, *n* = 19)). Overall response rate at day 100 post ASCT was 99% (CR 42%) with CR rate increasing to 62% at time of best response post transplant. Two year and 5 year overall survival rates were 90% and 67%, respectively, with an estimated median overall survival (OS) and progression-free survival (PFS) of 96 and 28 months, respectively. HRA was associated with a worse OS but not PFS (median OS: not reached for standard risk vs 60 months for HRA, *P* = 0.0006; median PFS: 27 months for standard risk vs 22 months for HRA, *P* = 0.70). The combination of VRd followed by ASCT is a highly effective regimen producing deep and durable responses in many patients.

## Introduction

There has been considerable progress in therapy for multiple myeloma in recent years. The introduction of novel agents including proteasome inhibitors and immunomodulatory drugs (IMiDs) have markedly improved response rates and survival^1,2^. Autologous stem cell transplantation (ASCT) has been standard therapy for multiple myeloma for over two decades with clinical trials showing an improvement in overall survival^3^. This is typically preceded by a period of induction therapy to rapidly control the disease in preparation for ASCT. A variety of induction regimens have been used over time, however, combinations including novel agents appear to be superior in a number of clinical trials^4–6^. The combination of bortezomib, lenalidomide, and dexamethasone was initially shown to be well tolerated and effective in a phase II study comparing it to other bortezomib based regimens^7,8^. The subsequent phase III SWOG S0777 trial confirmed its superiority in terms of response, progression-free, and overall survival when compared to patients treated with a combination of lenalidomide and dexamethasone^9^. Herein we report on outcomes of patients treated with a combination of bortezomib, lenalidomide, and dexamethasone followed by autologous stem cell transplant as upfront therapy for multiple myeloma at the Mayo Clinic.

## Methods

We conducted a retrospective review of all patients seen at Mayo Clinic Rochester that received induction with bortezomib, lenalidomide and dexamethasone followed by autologous stem cell transplantation for multiple myeloma, between 1st January 2010 and 30th of April 2017. Only patients transplanted within 12 months of diagnosis and receiving only one line of therapy prior to transplant were included in the study. The study was approved by the Mayo Clinic Institutional Review Board.

Risk stratification was according to the International Staging System (ISS) for multiple myeloma^10^. Limited data on lactate dehydrogenase (LDH) level at diagnosis were prior to 2015 which restricted our ability to stratify patients by the Revised International Staging System (RISS). High-risk cytogenetic abnormalities (HRA) were categorized in accordance with the International Myeloma Working Group (IMWG) definition and included deletion (17p), t(4;14), and t(14;16)^11^. Response and progression were defined according to consensus criteria published by the IMWG^12^. Response at day 100 was determined by assessing individual patient data of serum and urine protein electrophoresis, immunofixation, serum-free light chain assay, and bone marrow (BM) aspiration and biopsy obtained at ~100 days post ASCT. Deepening of response after this time point was assessed using serum and urine data as above without repeat BM aspiration and biopsy.

Statistical analysis was performed on JMP software (SAS, Cary, NC). Patient and disease-related factors were compared using the *χ*^2^ test for categorical variables, and the Wilcoxon signed rank test for continuous variables. Survival analysis was performed using the Kaplan–Meier method. Overall survival (OS) was calculated from date of ASCT to death from any cause. Progression-free survival (PFS) was defined as time to progression or death, calculated from date of ASCT. The Cox proportional hazards model was used to assess for predictors of OS. The variables included in the univariate analysis were age (≥65 vs <65), ISS stage (stage III vs stage I/II), cytogenetics (HRA vs standard risk), maintenance therapy (Maintenance/other therapy vs no maintenance) and best response post ASCT (<CR vs CR or stringent CR (sCR)). Variables reaching a *P* value < 0.1 were included in the multivariate analysis.

## Results

Baseline characteristics for the whole cohort (*n* = 243) are listed in Table 1 and are consistent with a transplant eligible population with multiple myeloma. HRA was present in 34% of patients with deletion (17p) detected in 21% of patients. The higher than expected number of patients with deletion (17p) is likely due to our institutional policy of considering velcade-based regimens for patients with HRA. Median follow-up time was 33 months post ASCT (95% CI: 28–38 months). The median time from diagnosis to transplant was 5 months (range: 2–12 months). Conditioning was full intensity melphalan 200 mg/m^2^ in 203 (84%) patients. Median duration of induction was 4 months (range: 1–10 months).

**Table 1:** Baseline characteristics whole cohort

Variable	Whole cohort (*n* = 243)
Age, median (IQR), years	61 (55–67)
Male, *n* (%)	151 (62)
ISS	
Stage I	72 (41)
Stage II	61 (34)
Stage III	44 (25)
Missing	66
FISH cytogenetics, *n* (%)	
High risk	70 (34)
Deletion(17p)	43 (21)
t(4;14)	19 (9)
t(14;16)	14 (7)
Missing	35
Time to transplant	
≤6 months	181 (74)
>6 months	62 (26)
Conditioning *n* (%)	
Melphalan 200 mg/m^2^	203 (84)
Melphalan 140 mg/m^2^	18 (7)
Carfilzomib/melphalan	17 (7)
Bortezomib/TBI/melphalan	3 (1)
BEAM	2 (<1)

*IQR* interquartile range, *ISS* international staging system, *FISH* fluorescent in situ hybridization, *TBI* total body irradiation, *BEAM* carmustine, etoposide, cytarabine, and melphalan

### Post-transplant therapy

Data on therapy post transplant were collected on all patients. Overall, 166 (68%) patients received some form of maintenance/other therapy post transplant. Four cohorts were identified, those receiving no maintenance (NM, *n* = 77), lenalidomide maintenance (LM, *n* = 108), bortezomib maintenance (BM, *n* = 39), and other therapy (OT, *n* = 19). Patients in the “other therapy” cohort received ongoing therapy post transplant with a variety of triplet or doublet regimens based on physician preference. Four patients received a tandem transplant, all of whom were in the OT cohort. Baseline characteristics for each cohort are listed in Table 2. The four cohorts were well matched for age, gender, and ISS stage. HRA was more common among patients receiving bortezomib maintenance or other therapy post transplant (NM 18% vs LM 22% vs BM 68% vs OT 79%, *P* < 0.0001). This was primarily accounted for by more frequent presence of deletion (17p) and t(4;14). Duration of induction and rates of full intensity melphalan conditioning were similar for all four cohort (median duration of induction: 4 months for NM vs 4 months for LM vs 4 months for BM vs 3 months for OT, *P* = 0.61; conditioning melphalan 200 mg/m^2^: 87% for NM vs 81% for LM vs 87% for BM vs 79% for OT, *P* = 0.56). Median duration of maintenance therapy was 12 months (IQR: 8–20 months) for lenalidomide and 15 months (IQR: 6–24 months) for bortezomib.

**Table 2:** Baseline characteristics by post-transplant therapy subgroup

Variable	No maintenance (*n* = 77)	Lenalidomide maintenance (*n* = 108)	Bortezomib maintenance (*n* = 39)	Other therapy (*n* = 19)	*P*-value
Age, median (IQR), years	60 (53–67)	62 (56–67)	61 (54–65)	58 (57–65)	0.68
Male, *n* (%)	50 (65)	68 (63)	22 (58)	11 (58)	0.81
Creatinine^a^, mg/dL, median (IQR)	0.9 (0.8–1.1)	1 (0.8–1.1)	0.9 (0.8–1.1)	0.8 (0.7–1.2)	0.56
Bone disease, n (%)	69 (89)	100 (92)	36 (92)	15 (80)	0.29
ISS, *n* (%)					0.19
Stage I	23 (43)	35 (41)	13 (48)	1 (9)	
Stage II	16 (30)	33 (38)	8 (30)	4 (36)	
Stage III	14 (27)	18 (21)	6 (22)	6 (54)	
Missing	24	22	12	8	
FISH cytogenetics, *n* (%)					
High risk	11 (18)	21 (22)	23 (68)	15 (79)	<0.0001
Deletion(17p)	6 (10)	13 (14)	13 (38)	11 (58)	<0.0001
t(4;14)	5 (8)	3 (3)	8 (24)	3 (16)	0.0034
t(14;16)	2 (3)	6 (6)	3 (9)	3 (16)	0.27
Missing	17	13	5	0	
Duration of Induction, median (IQR), months	4 (3–5)	4 (3–4)	4 (3–4)	3 (3–5)	0.61
Response to Induction, *n* (%)					0.12
≥VGPR	47 (61)	63 (58)	27 (69)	7 (37)	
≤PR	30 (39)	45 (42)	12 (31)	12 (63)	
Melphalan 200 mg/m^2^	67 (87)	87 (81)	34 (87)	15 (79)	0.56
Tandem transplant, *n* (%)	—	—	—	4 (21)	<0.0001
Duration of maintenance, median (IQR), months	—	12 (8–20)	15 (6–24)	—	0.61

*IQR* interquartile range, *ISS* international staging system, *FISH* fluorescent in situ hybridization, *VGPR* very good partial response, PR partial response

^a^Creatinine at time of transplant

### Hematologic response

**Fig. 1:**
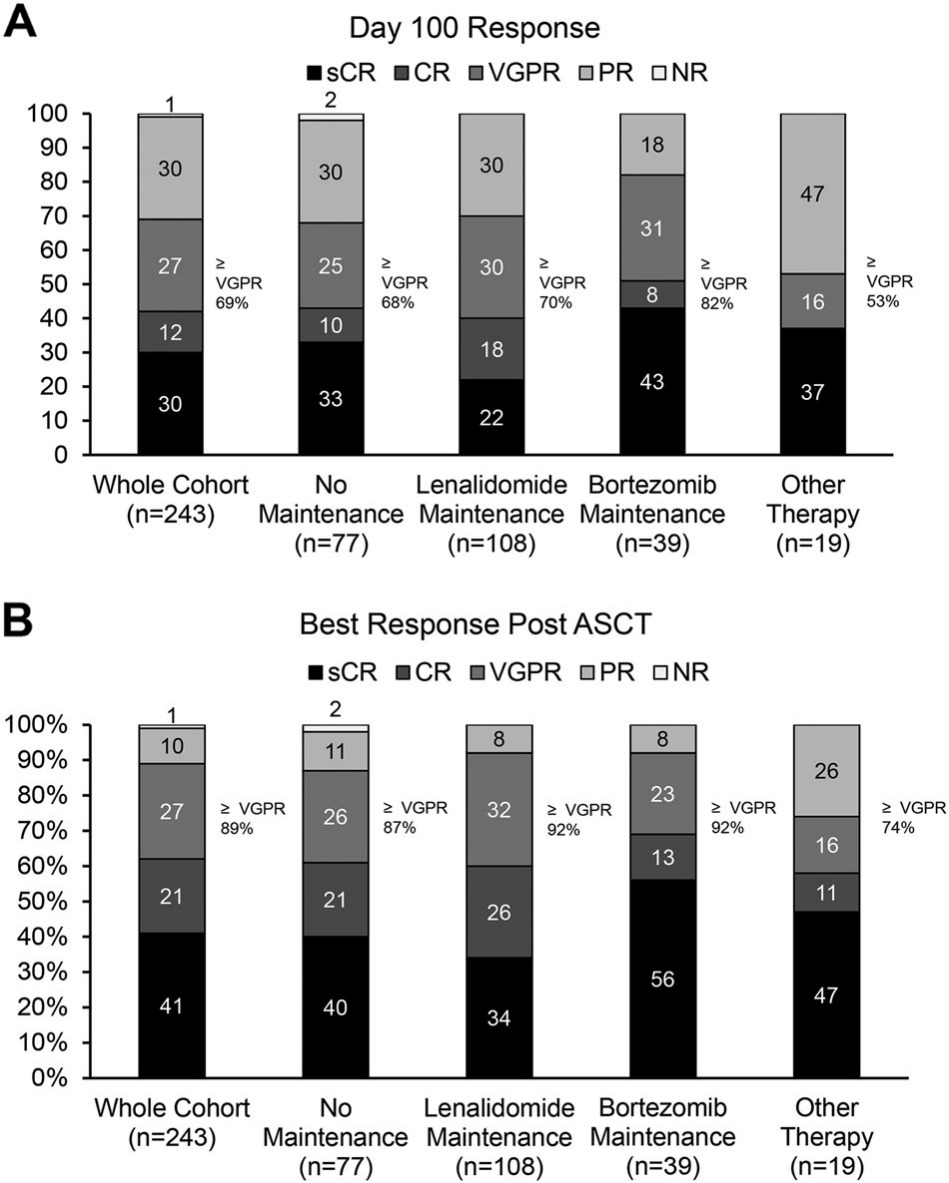
Response to therapy. Hematologic response at day 100 (**a**) and best response post transplant (**b**). sCR stringent complete response, CR complete response, VGPR very good partial response, PR partial response, NR no response

Data on response to therapy were available for all patients. Hematologic response at day 100 and best response post transplant for the whole cohort and each subgroup is summarized in Fig. 1. The overall response rate for the whole cohort was 99% at day 100 and at best response post transplant corresponding to a CR/sCR rate of 42% at day 100 and 62% at time of best response post transplant. ORR and the rate of CR/sCR were similar in all four subgroups (ORR: 97% for NM vs 100% for LM vs 100% for BM vs 100% for OT, *P* = 0.22; CR/sCR: 61% for NM vs 60% for LM vs 69% for VM vs 58% for OT, *P* = 0.76). There was a deepening of response between day 100 and best response post transplant in all four subgroups (≥VGPR rate: 68 to 87% for NM, 70 to 92% for LM, 82 to 92% for BM and 53 to 74% for OT). The median time to best response for patients not achieving best response by day 100 post ASCT was 11 months (IQR: 6–12 months).

### Survival

**Fig. 2:**
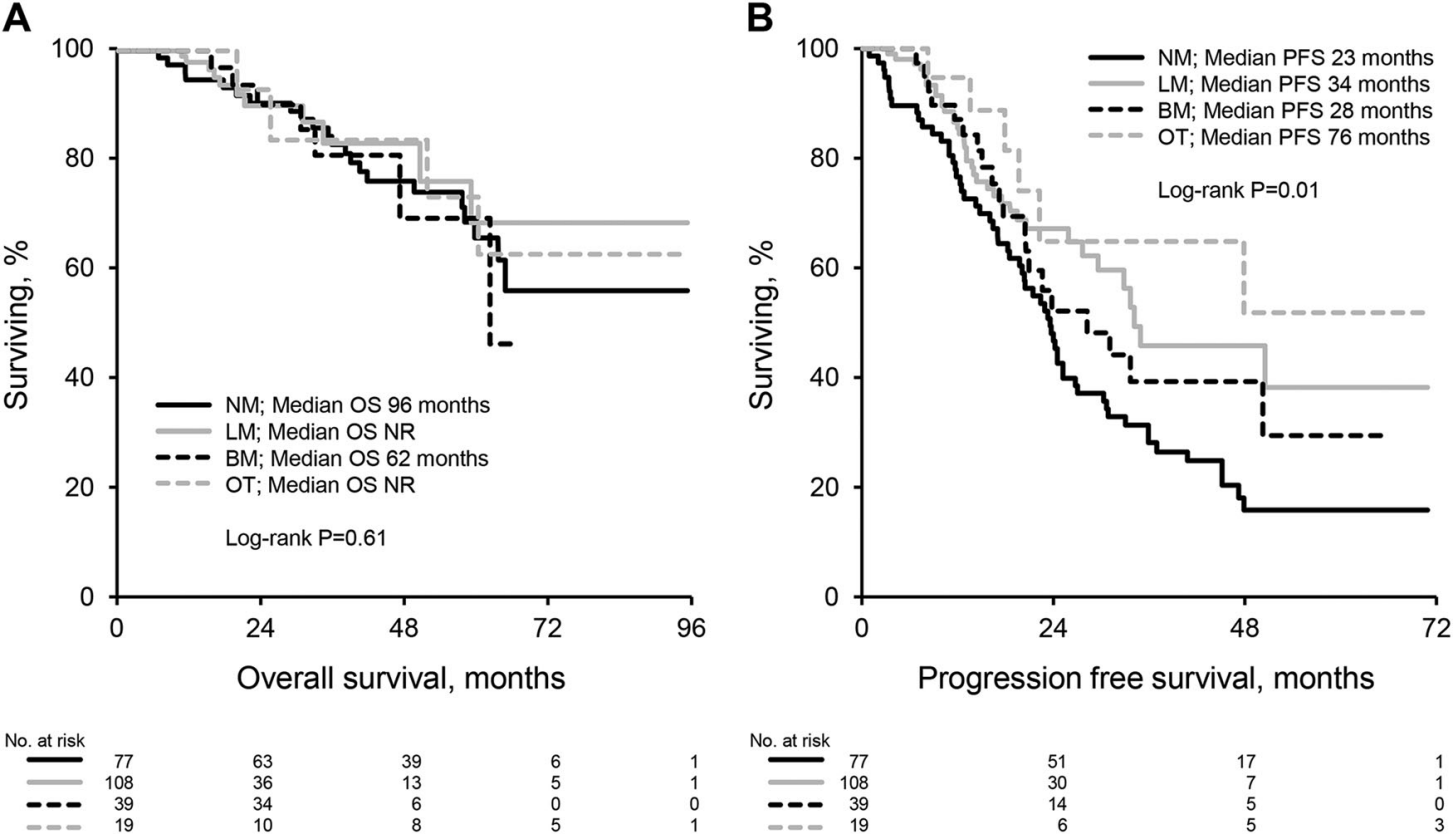
Overall and progression-free survival by post-transplant therapy. **a** Overall survival. **b** Progression-free survival. NM no maintenance, LM lenalidomide maintenance, BM bortezomib maintenance, OT other therapy, NR not reached

At last follow up 45 (19%) patients have died and 121 (50%) patients had relapsed. 2 year and 5 year overall survival rates were 90% and 67%, respectively. The estimated median OS and PFS for the entire cohort were 96 months and 28 months respectively. OS was not significantly different when stratified by post-transplant therapy (median OS 96 months for NM vs not reached for LM vs 62 months for BM vs not reached for OT, *P* = 0.61), however post-transplant therapy was predictive of PFS (median PFS 23 months for NM vs 34 months for LM vs 28 months for BM vs 76 months for OT, *P* = 0.01), Fig. 2. Compared to those receiving no maintenance, the difference in PFS was statistically significant only for patients receiving lenalidomide maintenance or other therapy. Patients receiving bortezomib maintenance had a similar PFS to those receiving no maintenance (median PFS 23 months for NM vs 28 months for BM, *P* = 0.15) and those receiving lenalidomide maintenance (median PFS 34 months for LM vs 28 months for BM, *P* = 0.49).

**Fig. 3:**
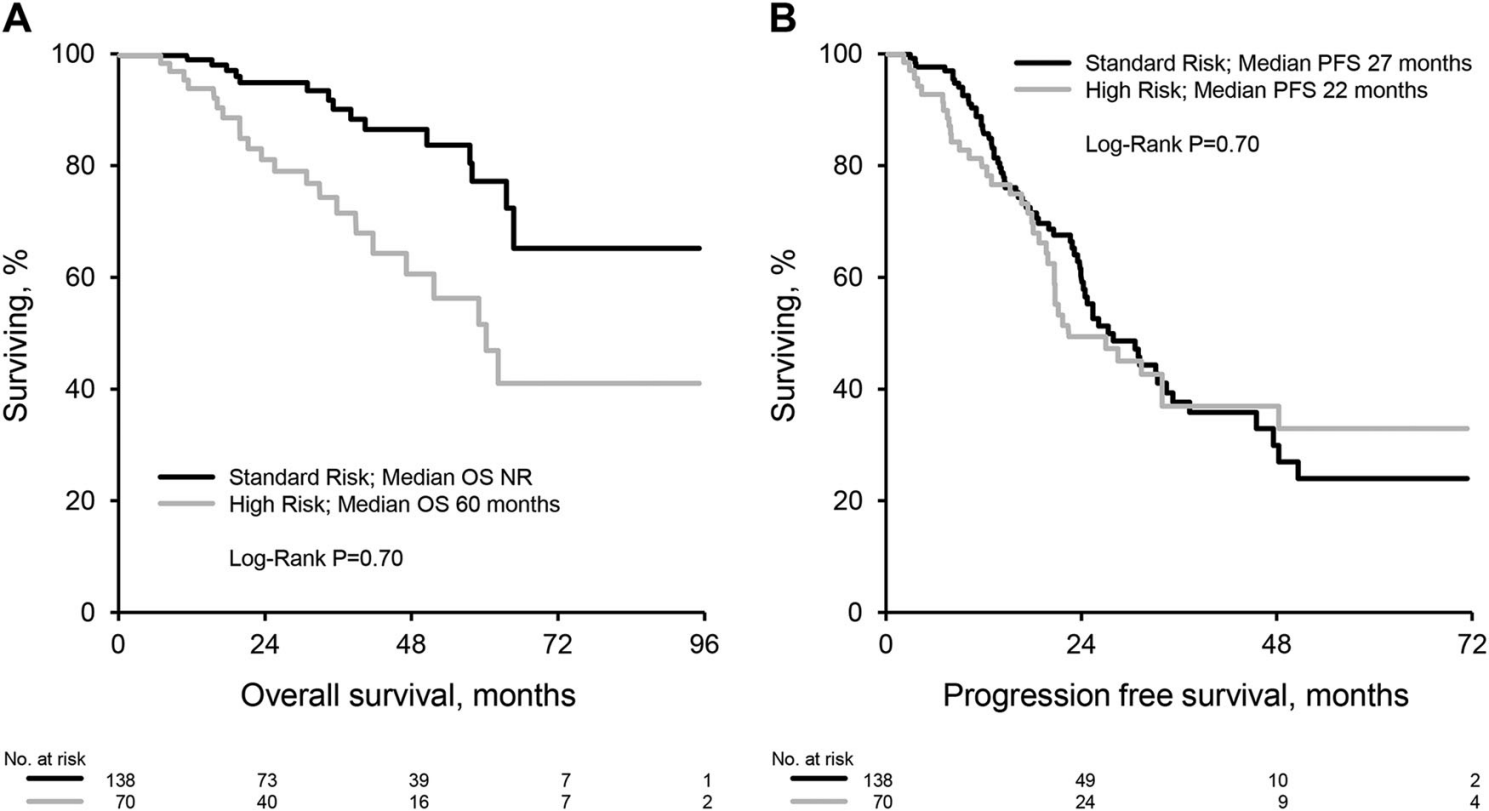
Survival by cytogenetics. **a** Overall survival. **b** Progression-free survival

**Fig. 4:**
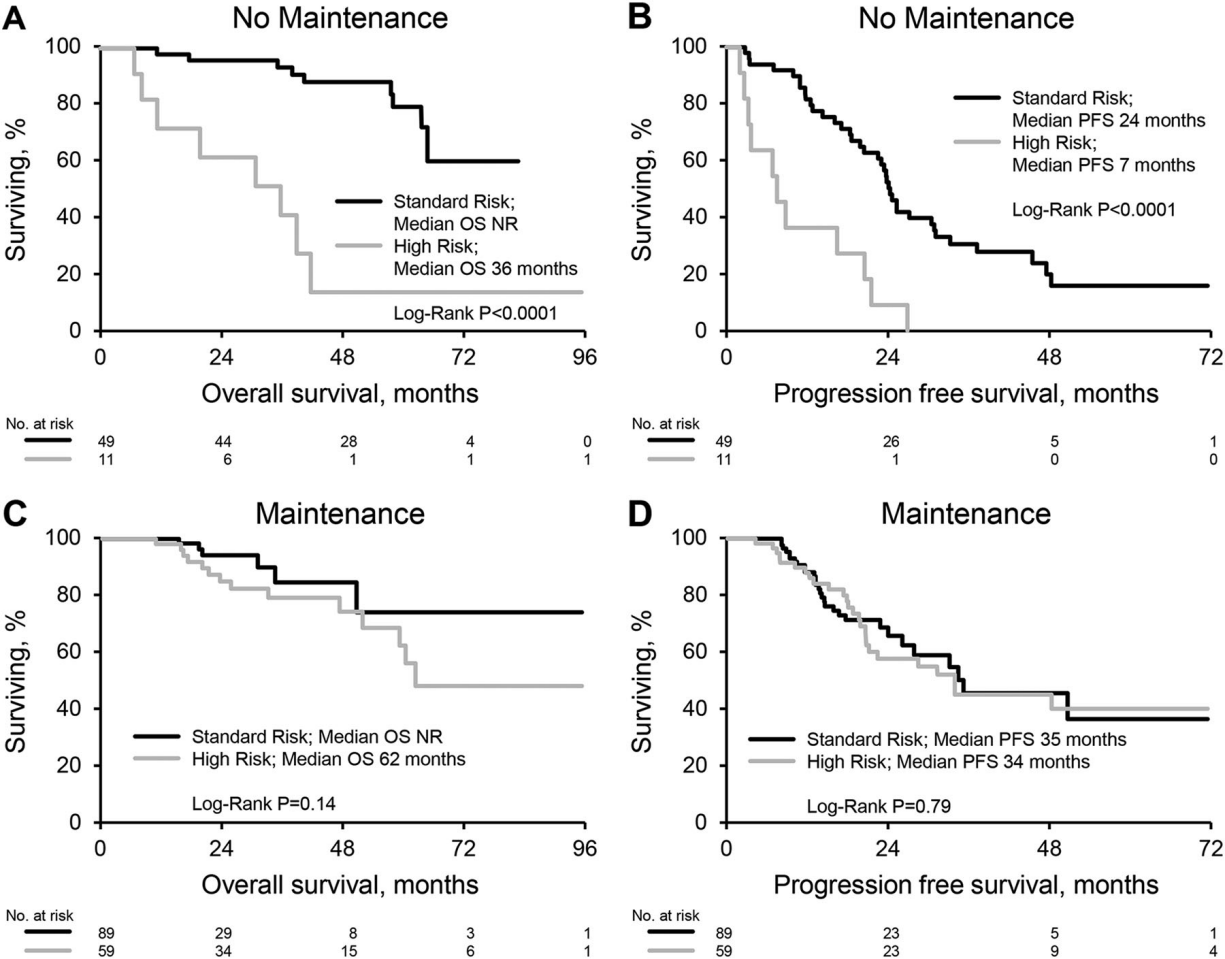
Survival by cytogenetics and post-transplant therapy. **a**, **b** Overall and progression-free survival by cytogenetic risk in patients receiving no maintenance. **c**, **d** Overall and progression-free survival by cytogenetic risk in patients receiving maintenance/other therapy

High-risk cytogenetics was associated with a worse OS but not PFS when compared to patients with standard risk (median OS: not reached for standard risk vs 60 months for HRA, *P* = 0.0006; median PFS: 27 months for standard risk vs 22 months for HRA, *P* = 0.70) (Fig. 3). In patients that did not receive maintenance therapy presence of HRA was a strong predictor of OS and PFS (median OS: not reached for standard risk vs 36 months for HRA, *P* < 0.0001; median PFS: 24 months for standard risk vs 7 months for HRA, *P* < 0.0001), Fig. 4. Patients receiving maintenance therapy appeared to have a similar PFS and OS irrespective of cytogenetics (median OS: not reached for standard risk vs 62 months for HRA, *P* = 0.14; median PFS: 35 months for standard risk vs 34 months for HRA, *P* = 0.79). The effect of maintenance/OT was variable when stratified by cytogenetic risk group. Among patients with standard risk maintenance/OT improved PFS (median PFS 35 months for maintenance/OT vs 24 months for NM, *P* < 0.05) but not OS (median OS not reached for maintenance/OT and NM cohorts, *P* = 0.97). Amongst patients with HRA maintenance/OT improved PFS (median PFS 34 months for maintenance/OT vs 7 months for NM, *P* < 0.0001) and OS (median OS 62 months for maintenance/OT vs 36 months for NM, *P* = 0.0006).

### Univariable and multivariable analysis

Results of univariable and multivariable models for PFS and OS are summarized in Table 3. On univariable analysis factors predicting PFS included male gender, maintenance/other therapy post transplant, and achieving CR/sCR. The only independent factors for PFS on multivariable analysis were ISS stage III and achieving CR/sCR. The only significant factors predicting OS on univariable and multivariable analysis were presence of HRA and achieving CR/sCR.

**Table 3:** Univariable and multivariable analysis

Variable	Progression-free survival				Overall survival			
	Univariable analysis		Multivariable analysis		Univariable analysis		Multivariable analysis	
	Hazard ratio (95% CI)	*P* value	Hazard ratio (95% CI)	*P* value	Hazard ratio (95% CI)	*P* value	Hazard ratio (95% CI)	*P* value
Age ≥ 65 vs <65	0.95 (0.63–1.38)	0.78	NA	NA	0.94 (0.48–1.77)	0.87	NA	NA
Male	1.48 (1.03–2.19)	0.03	1.26 (0.81–1.99)	0.31	1.11 (0.61–2.10)	0.74	NA	NA
ISS stage III vs I/II	1.55 (0.96–2.45)	0.07	1.75 (1.06–2.79)	0.02	1.88 (0.88–3.87)	0.1	2.12 (0.89–4.86)	0.08
HRA vs standard risk	1.08 (0.72–1.61)	0.7	NA	NA	2.99 (1.57–5.56)	0.0009	3.18 (1.38–7.76)	0.0063
Maintenance/other vs no maintenance	0.57 (0.39–0.81)	0.0021	0.73 (0.48–1.15)	0.18	0.86 (0.47–1.56)	0.61	NA	NA
CR/sCR vs <CR	0.46 (0.31–0.67)	<0.0001	0.43 (0.26–0.68)	0.0003	0.32 (0.17–0.59)	0.0002	0.11 (0.03–0.28)	<0.0001

*ISS* international staging system, *HRA* high-risk cytogenetic abnormalities, *sCR* stringent complete response, *CR* complete response, *NA* not applicable, *CI* confidence interval

## Discussion

We report outcomes of a large cohort of patients with multiple myeloma treated with a combination of bortezomib, lenalidomide and dexamethasone followed by ASCT in a non-trial setting. Our data show that induction with this regimen followed by ASCT within 12 months of diagnosis is highly effective therapy for myeloma with a hematological response at day 100 post transplant seen in 99% of patients with 69% achieving at least a VGPR. Response deepened overtime even amongst patients receiving no maintenance with 87% ultimately achieving at least a VGPR. This is comparable to results of a recent randomized trial of bortezomib, lenalidomide and dexamethasone with or without transplantation in which 78% of patients in the transplant group achieved at least a VGPR post transplant^13^. Deepening of response in a proportion of patients receiving no maintenance raises the question of optimal time of response assessment and decision making regarding maintenance or consolidation therapy. This may particularly be relevant for patients that clear their bone marrow of plasma cells but have persistent low level monoclonal protein detected on electrophoresis or immunofixation. Survival for multiple myeloma has significantly improved over time particularly in the era of novel agents^1,2^. The 5 year survival rate in our cohort of 67% with an estimated median overall survival of 96 months is encouraging and consistent with recent trends in improved survival for myeloma. We note however that follow up in a significant proportion of our patients is limited. Maintenance therapy in our cohort was prescribed according to physician preference. Patients with high-risk cytogenetic abnormalities were more likely to receive maintenance therapy with bortezomib or continue other therapies post transplant and this reflects our institutional recommendations to intensify therapy in this cohort beyond standard maintenance with lenalidomide. Maintenance lenalidomide therapy after stem cell transplantation has shown improved progression free survival with variable effects on overall survival in randomized clinical trials^14–16^. A meta-analysis of these trials suggested both a progression-free and overall survival benefit in patients receiving maintenance therapy with lenalidomide^17^. In our cohort, patients treated with lenalidomide maintenance had an improved progression free survival without a benefit in overall survival. Bortezomib therapy has been less well established as a maintenance regimen post ASCT. One study looking at bortezomib induction and maintenance in transplant eligible patients compared to non-bortezomib based induction and thalidomide maintenance showed improved progression free and overall survival in the bortezomib arm for the whole cohort as well as a subgroup analysis of patients with deletion (17p)^6^. In our study, patients receiving bortezomib maintenance were more likely to have high risk cytogenetics (68%), deletion (17p) (38%), and t(4;14) (24%). Despite this, progression free and overall survival for this group was no different to patients receiving no maintenance or lenalidomide maintenance. This may suggest the ability of bortezomib maintenance to overcome the poor prognostic impact of high risk cytogenetic abnormalities. Furthermore in our cohort patients with high risk cytogenetic abnormalities (who were more likely to receive consolidation (other therapy) or bortezomib based maintenance) had a similar PFS but worse OS compared to patients with standard risk. The similar PFS seen is likely due to the more intensive post-transplant therapy that this cohort received compared to patients with standard risk disease, who generally received lenalidomide maintenance or no maintenance therapy. The worse OS suggests that although progression is halted with intensive therapy post transplant, once these patients do progress, the underlying biology of the disease in these patients makes therapy in the relapsed setting less effective contributing to shorter survival. Myeloma therapy continues to progress rapidly however, and the second generation proteasome inhibitor carfilzomib has shown great promise and appears to be more potent than bortezomib. The combination of carfilzomib, lenalidomide, and dexamethasone has shown superior activity to lenalidomide and dexamethasone in relapsed multiple myeloma^18,19^. The results of a currently ongoing phase III trial comparing this regimen to bortezomib, lenalidomide and dexamethasone are eagerly awaited (NCT01863550).

Our study needs to be interpreted within the context of the limitations of a retrospective review. Response and assessment of progression after day 100 review was not uniform amongst all patients. Given we are a tertiary referral center many patients are monitored regularly at local institutions post transplant and return only intermittently for review. Data on response and progression collected at local institutions were not always available and this may influence our results on response, time to response and progression. Duration of induction therapy was variable. As an institution we typically proceed to transplant after 4 to 6 cycles of induction therapy in responding patients. However many patients are referred to us beyond 4 or 6 cycles of therapy and require a period of ongoing treatment whilst preparing for a stem cell transplant. Our guidelines, the Mayo Stratification for Myeloma And Risk-adapted Therapy (mSMART) published in 2013, recommended bortezomib based induction and maintenance for patients with high-risk cytogentics^20^. This selection bias is likely to account for the slightly higher than expected rate of deletion (17p) in our whole cohort and significantly greater proportion of patients with high-risk cytogenetics receiving bortezomib maintenance. We also note that patients had to receive stem cell transplantation to be included in our study. Patients receiving induction with bortezomib, lenalidomide, and dexamethasone who experienced significant toxicity or early mortality precluding them from consideration for stem cell transplantation, given the nature of our practice, may not have been referred to our institution and therefore excluded from our study.

Despite these limitations our study provides real world data on induction therapy with bortezomib, lenalidomide, and dexamethasone followed by stem cell transplantation showing this to be a highly efficacious approach for treatment of patients with multiple myeloma.
